# Late-onset rheumatoid arthritis registry study, LORIS study: study protocol and design

**DOI:** 10.1186/s41927-022-00322-7

**Published:** 2022-12-26

**Authors:** Masayo Kojima, Yutaka Kawahito, Takahiko Sugihara, Toshihisa Kojima, Ryozo Harada, Shintaro Hirata, Motomu Hashimoto, Toshihiko Hidaka, Hajime Ishikawa, Hiromu Ito, Mitsumasa Kishimoto, Yuko Kaneko, Kazuo Matsui, Toshihiro Matsui, Isao Matsushita, Akio Morinobu, Keiichiro Nishida, Eiichi Tanaka, Asami Abe, Michinori Ishitoku, Shuji Asai, Takashi Kida, Akira Onishi, Satoshi Takanashi, Masayoshi Harigai

**Affiliations:** 1 Nagoya City University, Kawasumi1, Mizuho, Nagoya, Aichi 466-8601 Japan; 2 National Center for Geriatrics and Gerontology, 7-430, Morioka-Cho, Obu City, Aichi 474-8511 Japan; 3 Inflammation and Immunology, Graduate School of Medical Science, Kyoto Prefectural University of Medicine, Kyoto, Kyoto Japan; 4 Division of Rheumatology and Allergology, St. Marianna University School of Medicine, Kawasaki, Kanagawa Japan; 5 Department of Orthopaedic Surgery, National Hospital Organization Nagoya Medical Center, Nagoya, Aichi Japan; 6 Department of Orthopaedic Surgery, Kurashiki Sweet Hospital, Kurashiki, Okayama Japan; 7 Department of Clinical Immunology and Rheumatology, Hiroshima University Hospital, Hiroshima, Hiroshima Japan; 8 Department of Clinical Immunology, Osaka Metropolitan University Graduate School of Medicine, Osaka, Osaka Japan; 9 Miyazaki-Zenjinkai Hospital, Miyazaki, Miyazaki Japan; 10 Department of Rheumatology, Niigata Rheumatic Center, Shibata, Niigata Japan; 11 Kurashiki Central Hospital, Kurashiki, Okayama Japan; 12 Department of Nephrology and Rheumatology, Kyorin University School of Medicine, Mitaka, Tokyo Japan; 13 Division of Rheumatology, Department of Internal Medicine, Keio University School of Medicine, Tokyo, Japan; 14 Department of Rheumatology, Teine Keijinkai Hospital, Sapporo, Hokkaido Japan; 15 Department of Rheumatology, National Hospital Organization Sagamihara National Hospital, Sagamihara, Kanagawa Japan; 16 Department of Rehabilitation Medicine, Kanazawa Medical University, Kanazawa, Ishikawa Japan; 17 Department of Rheumatology and Clinical Immunology, Graduate School of Medicine, Kyoto University, Kyoto, Japan; 18 Department of Orthopaedic Surgery, Science of Functional Recovery and Reconstruction Faculty of Medicine, Dentistry and Pharmaceutical Sciences, Okayama University, Okayama, Okayama Japan; 19 Division of Rheumatology, Department of Internal Medicine, Tokyo Women’s Medical University School of Medicine, Tokyo, Japan; 20 Department of Orthopaedic Surgery, Nagoya University Graduate School of Medicine, Nagoya, Aichi Japan; 21 Department of Clinical Immunology and Rheumatology, Hiroshima University Hospital, Hiroshima, Hiroshima Japan; 22 Department of Advanced Medicine for Rheumatic Diseases, Graduate School of Medicine, Kyoto University, Kyoto, Kyoto Japan; 23 Division of Rheumatology, Department of Internal Medicine, Keio University School of Medicine, Tokyo, Japan; 24 Division of Rheumatology, Department of Internal Medicine, Tokyo Women’s Medical University School of Medicine, Tokyo, Japan

**Keywords:** Prospective study, Elderly onset, Frailty, DMARD, MTX, Biologics, Patient and public involvement

## Abstract

**Background:**

Although drug treatment strategies for rheumatoid arthritis (RA) are relatively well established, there is a paucity of evidence on the treatment in older patients. The purpose of this study is to build a registry for late-onset RA (LORA), which is expected to increase rapidly worldwide. In addition, we aim to propose optimal treatment strategies according to the patient background including frailty, thereby contributing to improving the quality of treatment and daily living in patients with RA.

**Methods/design:**

The LORIS (Late-onset Rheumatoid Arthritis Registry) Study is a prospective nation-wide multicenter observational study of patients with LORA. The inclusion criteria were patients aged ≥ 65 years at onset, meeting 2010 ACR/EULAR classification criteria for RA, and starting either any disease-modifying antirheumatic drugs (DMARDs) in a DMARD-naïve patient or the first biologic/targeted synthetic DMARDs during the study period. Enrollment was started on 11 January, 2022 and will be closed on 31 December, 2023. Patients will undergo a comprehensive baseline assessment including clinical data, medication, cognitive and physical function, psychosocial factors, and frailty. Data will be collected at baseline, Month 3, 6, 12, 18, 24, 36, and summarized descriptively. The factors associated with adverse events and achieving remission will be determined.

**Discussion:**

A multi-disciplinary panel including patients, rheumatologists, and geriatric specialists will discuss the results and build a consensus regarding the treatment goals of LORA. We expect to provide a broad range of information for evidence-based shared decision making in the treatment of LORA.

*Study registration*: Registered at the UMIN registry (UMIN000046086) on 1 January 2022.

**Supplementary Information:**

The online version contains supplementary material available at 10.1186/s41927-022-00322-7.

## Background

Rheumatoid arthritis (RA) is a typical chronic autoimmune disease, where activities of daily living (ADL) will be gradually restricted owing to joint dysfunction. Currently, it is estimated that more than 800,000 patients with RA are treated in Japan, of which the late elderly patients account for 35% [1, 2]. Conventionally, RA has been considered to most commonly occur in people in their 30 s–50 s; however, people who developed RA in their late 60 s have increased in number in recent years [3]. With the advent of super-aging society, it is plausible that the number of patients with late-onset RA (LORA) increase rapidly and the establishment of treatment strategy for LORA is an urgent issue.

Drug therapy for RA has dramatically evolved in the twenty-first century, and joint destruction can now be prevented by early initiation of thorough anti-inflammatory treatment [4, 5]. In 1999, methotrexate (MTX) was approved as a therapeutic drug for RA in Japan. In 2003, infliximab, the first biologic DMARD (bDMARD), was approved; and now, 11 different bDMARDs including biosimilars can be prescribed. In addition, starting with tofacitinib in 2013, five types of Janus kinase (JAK) inhibitors have been approved, and treatment options have become remarkably wider compared to 20 years ago.

In 2020, the Japan College of Rheumatology (JCR) updated the clinical practice guidelines for the management of RA [6]. This set of guidelines included recommendations for elderly RA based on a systematic review of drug therapy, and the use of MTX and molecular targeted agents were weakly recommended with careful safety considerations [7]. However, the quality of evidence was very low because it was based on sub-analyses of RCTs. Safety information is not always reported adequately in RCT studies [8]. Lack of an evidence-based treatment strategy for elderly patients may cause insufficient control of disease activity, leading to progression of joint destruction, decreased ADL, and increased incidence of RA-related comorbidities including secondary osteoporosis and cardiovascular events.

The elderly tends to have many comorbidities and attending physicians may become overly cautious about possible adverse events (AEs) and miss the opportunities for treatment [9, 10]. Actually, based on the analysis of Nakajima et al*.* using the National Database of Health Insurance Claims [2], the proportion of patients using MTX, the anchor drug for RA treatment, decreases with age; 73% of patients in their 50 s and 38.2% in those aged ≥ 85 years. In addition, bDMARDs are administered at 24% in the 50 s, but only at 13.7% among patients aged ≥ 85 years. Meanwhile, the oral corticosteroids are used at 37.3% in the 50 s, but as high as 52.0% at 85 years or above. However, previous studies showed inconsistent results in the association between age and AEs, thereby indicating that advanced age alone is not necessarily a significant risk factor of serious AEs in RA patients treated with DMARDs. Recently, Sugihara et al*.* has reported that age was not a significant risk factor of serious AEs after adjusting for comorbidities at baseline and mean SDAI during the observation period and concluded that T2T strategy by using MTX and bDMARDs was effective and feasible for the LORA [11]. Based on retrospective cohort data in patients with RA who started treatment with biologic / targeted synthetic b/tsDMARDs, Ochi et al. reported that safety and therapeutic effectiveness were comparable between late-onset and non-late-onset cases [12].

Frailty is a concept that reflects the limited mental and physical reserve capacities of the elderly [13, 14]. In general, the elderly require treatment tailored to their biological age, which takes into account the complications of individual patients and the comprehensive functional assessment of the body and mind, that is, the severity of frailty [9, 10]. Frailty assessment at the start of treatment may guide the attending physician’s treatment selection and play a role in prognosis prediction and improvement of patient QOL [15].

Increasing medical costs due to the growing elderly population have become a social issue. b/tsDMARDs are expensive so that some patients reluctant to start. Considering the loss of workforce and care costs due to decline of ADL, early and appropriate use of b/tsDMARDs for older patients may contribute to the improvement of the health economic aspects of the problem.

Thus, prospective observational studies are necessary to confirm the safety and effectiveness of the treatment for LORA.

The objective of this study is: (1) building a registry for LORA; (2) performing a comprehensive functional assessment (i.e., physical aspects including frailty, mental and cognitive aspects, and social aspects); (3) proposing optimal treatment strategies tailored to the patient background; and (4) contributing to quality improvement of medical treatment for RA in Japan and extension of healthy life expectancy. This study is conducted to generate evidence to update the JCR clinical practice guidelines in collaborations with the JCR and the Japan Rheumatism Friendship Association [16].

## Methods/design

### Study design

The LORIS (**L**ate-**o**nset **r**heumatoid arthr**i**tis regi**s**try) Study is a prospective, nation-wide, multicenter, observational study of patients with LORA. A total of 19 leading medical institutions of RA (10 university hospitals, two national hospitals, one municipal hospital, three private hospitals, and one clinic) were requested by the steering committee to join the study as the study centers. Additional hospitals or clinic may join the study when the representatives of any study centers recommend, and the steering committee approve them.

### Participants

Participants developing RA at 65 years or older, fulfilling the 1987 American College of Rheumatology classification criteria for RA or the 2010 ACR/EULAR criteria, and who meet one of the followings are enrolled: (1) First therapy with any csDMARD is initiated, (2) First MTX treatment is initiated, or (3) First b/tsDMARDs treatment is initiated during the study period (i.e., from January 10, 2022, to December 31, 2023).

Exclusion criteria are as follows: (1) Patients who are unable or unwilling to provide written informed consent, (2) Those who have already taken any b/tsDMARDs previously, (3) Patients with any other systemic autoimmune disorders, except for Sjogren’s disease, (4) Patients who are deemed inappropriate for enrollment by the investigators, and 5) Patients in whom more than two years have passed since the diagnosis was confirmed.

### Patient and public involvement

Two patient representatives of the Japan Rheumatism Friendship Association [16] participated in the panel meeting to determine the study protocol on December 9, 2021. They are expected to join the second panel meeting which is planned in March 2024 to discuss the study results and build a consensus of treatment goals for LORA.

### Outcomes

We have two primary outcomes for this study: the safety of the treatment and changes in QOL. Specifically, we will identify followings.

1. Factors related to survival and cause of death
2. Factors related to hospitalization (malignancy, infection, cardiovascular events, fracture and pneumonia)
3. Prevalence of requiring long-term care and their grade
4. Changes in QOL (EQ-5D-5L) and factors related to the decline in QOL
5. Changes in physical function (HAQ) and factors related to decline in physical function

Secondary outcomes include the treatment effectiveness and drug retention rates of the initial DMARD. We will assess proportion of those who achieve sustained clinical remission and low disease activity, and achievement of minimally clinically important difference in physical function (HAQ) and QOL (EQ5D). Sustained remission is defined as a Disease Activity Score in 28 joints (DAS28) of < 2.6 for 6 months (short-term sustained remission) or for 24, and 36 months (long-term sustained remission).

We also examine followings.

1. Trends in frailty and sarcopenia
2. Changes in physical functions other than HAQ and factors related to the decline in physical functions
3. Changes in cognitive function and factors related to decline in cognitive function
4. Changes in disease activity
5. Changes in joint destruction
6. Changes in Patient-reported Outcome
7. Whether MTX is administered, the dose and interval, if not, reasons for dose increase, decrease, or discontinuation
8. Whether or not molecularly targeted drugs are administered, and reasons for prolonging the interval, reducing the dose, or discontinuing.
9. GCs administration or not, dose, and reasons for dose increase or decrease or discontinuation
10. Whether or not NSAIDs are administered.

### Participant timeline

Enrollment was started January 10, 2022, and will be closed on December 31, 2023. Patients will be followed for at least three years after enrollment.

### Sample size

Given that the LORIS Study is a longitudinal observational study to describe the treatment patterns of LORA, the targeted sample size was not determined. A larger sample size increases the data confidence. According to the past record of each institution, it is expected that approximately 600–700 patients will be recruited.

### Recruitment

Patients who visit the study centers are requested to participate in the study by the attending rheumatologists if they are eligible based on the inclusion / exclusion criteria described above. Participants receive usual care according to site investigators’ discretion without any intervention from the study protocol.

### Data collection and management

After providing written informed consent, patients are enrolled in the registry. Clinical assessment data including physical examination, laboratory tests, and details of medication are recorded in the electronic data capture (EDC) system at each study center. The coordinating center will manage the EDC system.

Patients are asked to complete questionnaires to capture information regarding demographics and patient-reported outcomes. Present and past medical history are collected by nurses or trained clinical research coordinators and confirmed by site investigators. Clinical assessment data will be collected at baseline, Month 3, 6, 12, 18, 24, and 36. Questionnaires and interview surveys will be conducted at baseline, Month 12, 24, and 36. Detailed information of data collection is shown in Table 1.

**Table 1:** Parameters captured by the registry database

Category	Items
Sociodemographic data	Age, sex, education, marital status
Medical history	Onset time Current symptoms of RA compared to those in the previous year Surgery for RA, site, type Total number of medications taken Vaccination history (*Streptococcus pneumoniae*, herpes zoster)
Medication	Use/non-use of MTX, start date, dose, reason for non-use (cognitive decline, interstitial pneumonia (IP), pulmonary diseases other than IP, renal dysfunction, hepatic dysfunction, history of infection, peripheral blood test results, complication/history of malignant tumor, and HBV carrier/infection, among others) Use/non-use of other csDMARDs Use/non-use of GCs, dose, administration period, and reason for administration Use/non-use of NSAIDs Use/non-use of opioids Use/non-use of molecular targeted drugs, start date, and dose Use/non-use of osteoporosis drugs (bisphosphonates, PTH preparations, denosumab, romosozumab, vitamin D preparations, and SERM)
Symptoms	Tender joint count (28・44) Swelling joint count (28・44) Physician global assessment (VAS) Patient global assessment (VAS) Physical function (HAQ-DI) Health-related QOL (EQ-5D-5L) Depression (PHQ-2) Pain and fatigue (VAS)
Laboratory data	Inflammation marker (CRP, MMP3, ESR) Blood cell count (White blood cell, neutrophil, total lymphocyte, red blood cell, hemoglobin, and platelet) Nutritional marker (Serum total protein, serum albumin, Hemoglobin A1c, serum total cholesterol, LDL, HDL cholesterol, TG) Infection tests (HBs antigen, HBs antibody, HBc antibody, HCV antibody, IGRA, and β-D-glucan) Autoimmune tests (rheumatoid factor, antinuclear antibody, anti-SS-A/Ro antibody, and anti-CCP antibody) Renal function (Serum creatinine, Serum cystatin C, eGFR-Cre) Liver function (AST or ALT) 25-hydroxy vitamin D (25OHD) Krebs von den Lungen-6 (KL-6)
Radiological examination	Plain X-ray (Xp) imaging (frontal and lateral chest, both upper and lower limbs, or all frontal images). The modified Sharp score will be calculated every year to assess the progression of joint destruction Bone density (DXA method, lumbar spine, or mean value for both sites)
Comorbidity	Malignant tumor (solid cancer, hematological cancer, among others, the presence or absence of metastasis) Lung disease (IP, COPD, pulmonary tuberculosis, atypical mycobacteriosis, bronchiectasis, or bronchiolitis, among others) Heart disease (myocardial infarction, other cardiovascular disease, chronic heart failure, or peripheral vascular disease, among others) Stroke Hemiplegia or paraplegia Hypertension Diabetes mellitus Hyperlipidemia (hypo-HDL-emia (< 40 mg/dl), hyper-LDL-emia (≥ 140 mg/dl), hypertriglyceridemia (≥ 150 mg/dl) Fracture (spine or femur, among others) Depression Gastrointestinal disease (peptic ulcer or colonic diverticulitis) Liver disease (hepatitis B, hepatitis C, or liver cirrhosis, among other liver dysfunctions) Renal disease (dialysis, decreased renal function [eGFR < 60]) Osteoarthritis (DIP, shoulder, elbow, knee, or hip joints) Herpes zoster Venous thromboembolism (pulmonary thromboembolism or deep vein thrombosis)
Lifestyle and health behavior	Smoking status Physical disability certification and the grade Certification of long-term care needs and the care level Social participation status Exercise habits
Physical examination	Height and body weight Grip strength Body composition (only at institutions where it is possible) Walk speed (only at institutions where it is possible, 10 m at comfortable walking speed) The five-times sit-to-stand test (only at institutions where it is possible)
Cognitive assessment	MMSE [17] will be performed for cognitive function assessment. It will be implemented on patients aged ≥ 75 years at enrollment, who are starting csDMARDs for the first time in this study, and when treatment contents are changed due to cognitive decline
Frailty assessment	Physical frailty based on J-CHS criteria [18] Kihon checklist [19–22] Questionnaire for medical checkup of old-old [23] Clinical frailty scale [24]

We will stop observation of the patient when he or she requests to withdraw from participation in the study, if there is no visit to the hospital for more than 6 months, or when the principal investigator determines that it is difficult to continue observation.

Enrollees are allowed to withdraw anytime. However, the data collected before consent withdrawal cannot be deleted and data of such enrollees will be analyzed or will be used secondarily.

Personal information enabling direct identification of any individual will only be handled by each research center. All data are sent to the study coordinator after anonymization.

The protocol of this study has been registered to the public clinical trial registration database UMIN-CTR (UMIN000046086) before the start of this study. Study results will also be updated and reported on the database as needed.

### Statistical methods

As registry implementation status, numbers of enrolled individuals, of patients whose data are obtained at each survey period, and of patients who have discontinued will be shown.

For variables related to patient background and treatment status, distribution will be shown by mean with standard deviation, median with IQR, or number and proportion in contingency tables.

The information of serious AEs and person-year will be obtained, and the survival rate with the 95% confidence intervals by treatment options will be estimated by person-year method. Serious AE requiring hospitalization, infectious AE, AE leading to death will be analyzed separately. Impact of baseline variables on each outcome will be investigated using multivariable analysis. A two-tailed *p* < 0.05 will be considered statistically significant. In addition, comparison between the age groups < 75 years old and ≥ 75 years old will be performed.

### Consensus building

Based on the findings of the registry data analysis, consensus regarding treatment strategies of LORA will be built by using the modified Delphi process. Panel meetings consisting of the steering committee members, the representatives from each research centers, geriatricians, the experts of data management and RA patient representatives will be held to discuss about treatment strategies and goals. Eighty percent agreement within three rounds of discussion and voting is necessary to achieve consensus.

### Dissemination policy

The results of this study will be widely disseminated at domestic and overseas academic conferences, and in scientific papers. In addition, the results will be reflected in the JCR Clinical Practice Guidelines for the management of RA scheduled to be revised in 2024.

## Discussion

In LORA patients, both physicians and patient themselves tend to become overly cautious to AEs of DMARDs, which may lose opportunities to recover their ADL and QOL. It is well recognized that a considerable degree of caution is required when administering MTX to the elderly. Owing to the declining renal function with age, older patients may have a higher risk of complications due to MTX use [7]. Guidelines for MTX treatment have recommended starting at a low dose and setting a lower maximum dose for elderly patients but have not provided a clear criterion. Liver disorders, stomatitis, gastrointestinal disorders, cytopenia, and infections increase in a dose-dependent manner [25]. In the elderly patients with RA, the risk of developing lymphoproliferative disorders associated with MTX use is also known to increase [26]. In this study, we will monitor LORA patients who are starting MTX treatment carefully and determine the conditions in which treatment can be continued safely. We will also examine the factors that hamper continuing treatment and clarify the condition with which MTX are used appropriately.

As for b/tsDMARDs we have reported high patients’ satisfaction by conducting interview and questionnaire surveys [27, 28]. In these studies, some concerns regarding high medical costs as well as the increased risks of infection and malignancy have been disclosed. In the current study, we will compare the differences in patient characteristics AEs by treatment. It will clarify the appropriate conditions to use b/tsDMARDs.

Corticosteroids can be used even in patients who cannot tolerate DMARDs due to decreased renal function and other comorbidities, and it has been confirmed that the proportion of patients with prescription of corticosteroids tends to increase with age [29]. The minimum use of short-term corticosteroids in combination with csDMARDs in older patients with active early RA was weakly recommended by the guideline panels of the 2020 JCR guidelines [6]. Continuous use of corticosteroids causes osteoporosis, which directly lead to bone fractures, hospitalization, and the need for nursing care. Increased risk of severe infection due to corticosteroid use should also be warned. In this study, the use of corticosteroids and relevant patient background will be examined in detail to clarify acceptable dose and period of corticosteroids use in LORA.

### Limitations

Because of the observational study design, there are no comparator treatment group, which could make analyses of effectiveness and safety of treatments difficult. The evidence level obtained from this study will be evaluated low or very low by the Assessment, Development and Evaluation (GRADE) system. This study group is mainly comprised of the committee members of the 2020 JCR clinical practice guidelines for RA. The participants of this study will receive treatment from the expert rheumatologists so that treatment outcomes obtained in this study are expected to be superior to those at general medical facilities. In the absence of evidence regarding initial treatment for LORA, the elucidation of the level of prognosis that can be expected in the case of ideal treatment is essential to setting target values for RA treatment in Japan. To disseminate our findings to general outpatient clinics, we must need identifying the barriers for implementation (Additional file 1).

## Supplementary Information

**Additional file 1**. A plain language summary of the LORIS Study in English and Japanese.

## Data Availability

The principal investigator of each research center keeps a record of data sharing with the joint research centers. When a third-party institution requests data sharing, the steering committee of this study shall review the study protocol and determine the permission and scope of data sharing.

## Abbreviations

ADL: Activities of daily living
AE: Adverse event
DMARD: Disease-modifying antirheumatic drugs
EDC: Electronic data capture
IP: Interstitial pneumonia
LORA: Late-onset rheumatoid arthritis
LORIS: Late-onset rheumatoid arthritis registry
QMCOO: Questionnaire for medical checkup of old-old
RA: Rheumatoid arthritis
RCT: Randomized controlled trials
