# Supplementary material for: Late-onset rheumatoid arthritis registry study, LORIS study: study protocol and design
Source: BMC Rheumatol. 2022 Dec 26;6:90. doi: 10.1186/s41927-022-00322-7 (PMC9791765; doi:10.1186/s41927-022-00322-7)
Supplement: Supplementary file 1 — Additional file 1. A plain language summary of the LORIS Study in English and Japanese. [file 41927_2022_322_MOESM1_ESM.docx]

A plain language summary of the LORIS Study

<English>

1. Background and purpose of this Study

Drug therapy for rheumatoid arthritis (RA) has improved dramatically since the beginning of the 21st century. However, the evidence for the treatment of RA is mainly based on data for patients under 65 years of age. On the other hand, the number of cases occurring at an older age has been increasing in recent years.

The purpose of this study is to investigate in detail what kind of treatment is used for elderly patients with RA, how well it works, and how safe it is. Through this study, we aim to propose optimal treatment methods tailored to the background of each patient and contribute to improving the quality of rheumatology care in Japan and extending healthy life expectancy of RA patients.

1. Who can participate?

Patients 65 years of age or older who have developed RA diagnosed by a rheumatologist and have started treatment with either synthetic anti-rheumatic drugs or molecular targeted drugs are eligible to participate.

1. Research Flow

Study period: September 7, 2021 - March 31, 2028

Study enrollment period: January 2022 - December 2023

Post-registration follow-up period: 3 years after registration

　In this study, questionnaire responses and information on treatment will be collected at 3 months, 6 months, 1 year, 1.5 years, and annually thereafter from the time of initial enrollment.

The information to be collected is as follows.

Medical information: date of birth, gender, date of diagnosis, drugs used, subjective and objective findings, laboratory values, medical history and complications, radiological examinations, surgical history, height, and weight.

Questionnaire: Questions regarding subjective health perspective, social participation status, depression, educational background, family structure, exercise habits, frailty, and sarcopenia

Physical and cognitive function measurements (only at selected facilities): grip strength, body composition, walking speed, five times standing and sitting, cognitive function assessment

1. Publication of study results

The results obtained from this research will be presented at domestic and international conferences, academic journals, etc. Information that can identify individuals will not be disclosed.

1. Fundings and conflict of interest

This research is supported by the Japan Agency for Medical Research and Development (AMED) and the Research Project Research Fund and the Longevity Medical Care Research and Development Fund. Prior to the implementation of this research, the researchers conducting this research has reported the status of conflicts of interest in this research, including personal earnings, to ensure transparency.

A detailed description of this research and the status of conflicts of interest at each research institution are available on the research group's website (https://www.ncgg.go.jp/ri/lab/cgss/LORA-registry/).

<Japanese>

**1　本研究の目的・意義**

関節リウマチ（リウマチ）の薬物治療は21世紀に入り飛躍的に向上し、治療方法が確立されてきました。しかしながら、その科学的根拠は主として65歳以下を対象としたデータに基づいており、65歳以上の患者さんの初期治療に関する科学的根拠は十分確立されていません。一方、近年高齢での発症例が増えており、団塊の世代ジュニアの高齢化に伴い、今後は益々高齢発症のリウマチ患者さんが急増すると考えられます。

本研究は、高齢発症のリウマチ患者さんを対象として、どのような治療が行われ、どのくらいの効果が得られているのか、安全性はどうか、などを詳しく調べることを目的としています。本研究により、患者さんそれぞれの背景に合わせた最適な治療方法を提案し、わが国のリウマチ診療の質の向上と健康寿命の延伸に貢献することを目指しています。

**2　ご参加いただける人**

65歳以上で関節リウマチを発症し、リウマチ専門医による確定診断を受け、合成型抗リウマチ薬、分子標的薬いずれかの薬物治療を開始した人が対象となります。

**3　研究の流れ**

研究期間：2021年9月7日～2028年3月31日

研究登録期間：2022年1月～2023年12月

登録後の追跡期間：登録後から3年

　本研究では、アンケートのご回答と治療に関する情報等を、初回登録時から3か月、6か月、1年後、1年半後、以降は年に1 回、収集します。

情報収集する内容は以下のとおりです。

診療情報：生年月、性別、診断年月、使用薬剤、自覚・他覚所見、臨床検査値、既往歴・合併症、放射線検査、手術歴、身長、体重

アンケート：主観的健康観、社会参加状況、抑うつ、学歴、家族構成、運動習慣、フレイル、サルコペニアに関する質問

身体機能測定・認知機能（一部施設のみ）：握力、体組成、歩行速度、5回立ち座り、認知機能評価

**4　研究結果の公開**

**この研究により得られた結果は、国内外の学会や学術雑誌等で、発表する場合がありますが、個人が特定できる情報は公開しません。**

**5　運営資金・利益相反**

本研究は、日本医療研究開発機構（AMED）の研究事業研究費、長寿医療研究開発費により実施・運営されています。この研究を行う研究者は、この研究の実施に先立ち、個人の収益等、この研究の利益相反に関する状況について研究機関の長に報告し、透明性を確保しています。

本研究の詳しい説明、各研究機関における利益相反の状況は、研究班ホームページ（https://www.ncgg.go.jp/ri/lab/cgss/LORA-registry/）上で公開されています。
